# Influence of Building Direction on the Oxidation Behavior of Inconel 718 Alloy Fabricated by Additive Manufacture of Electron Beam Melting

**DOI:** 10.3390/ma11122549

**Published:** 2018-12-14

**Authors:** Lv Li, Xiaojuan Gong, Xianjue Ye, Jianwei Teng, Yan Nie, Yunping Li, Qian Lei

**Affiliations:** 1 School of Materials Science and Engineering, Central South University, Changsha 410083, China; 163112095@csu.edu.cn; 2 State Key Lab for Powder Metallurgy, Central South University, Changsha 410083, China; xiaojuangong@csu.edu.cn (X.G.); yexianjue@csu.edu.cn (X.Y.); tengjianwei@csu.edu.cn (J.T.); 3 Yuanmeng Precision Technology (Shenzhen) Institute, Shenzhen 518055, China; nieyan725@163.com

**Keywords:** Inconel 718 alloy, oxidation behavior, electron beam melting (EBM), diffusion

## Abstract

This research was aimed at investigating the high temperature oxidation behavior of Inconel 718 superalloy fabricated by electron beam melting with the building direction of 0°, 55° and 90° deviation from the Z axis of cylindrical samples. Columnar γ-fcc phase with preferred crystal orientations was found in all specimens. With the temperature ranging from 700 to 1000 °C, the 0° sample, symbolized by the lowest grain boundary density, and largest grain size, reveals the best oxidation performance. It is concluded that the building direction has more impact on the amount of grain boundary density than crystal orientation, which determined the oxidation resistance.

## 1. Introduction

Nickel-based superalloys are a kind of precipitate hardening alloys, predominantly strengthened by γ′ phase (Ni_3_(Al, Ti, Nb), *L*12) and/or γ″ phase (Ni_3_Nb, *D*022) [1], and have been extensively applied in the aerospace industry, especially in the hot sections of aero turbine engines, due to their great high-temperature strength and extraordinary corrosion resistance etc. [2,3]. Nickel-based superalloys have been manufactured in a large number of methods, such as wrought, cast, and powder metallurgy (normally with Hot Isostatic Pressing (HIP) treatment) etc., and can give rise to satisfactory performances in various aspects [4,5,6,7,8]. With the rapid development of the aerospace industry, higher temperature and more reliable Inconel 718 components of the modern industry is in great demand. Poor oxidation resistance of any thermo-resistance component can cause a potential risk to its service reliability leading to a shorter service life. Hence, degradation by high temperature oxidation is a main reason for the failure of hot-section turbine blades [9]. Additionally, the need for complex shapes of turbine blades is increasing. Consequently, the net shape forming methods, in particular the 3D printing technologies or also called additive manufacturing (AM) techniques, have attracted the attention of manufacturers [10].

Compared to the traditional AM technique of selected laser melting (SLM), electron beam melting (EBM) is particularly famous for its flexible abilities to build different sections with fewer defects or cracks because of its high-power and fast scanning electron beam [11]. During this process, the materials are deposited layer-by-layer followed by a rapid cooling, giving rise to different microstructures and mechanical properties compared to the conventional methods. Besides, the preheating process is unique for the EBM process, due to uneven temperature distribution of the forming zone and electric charge excessively accumulating on metal powder surface, the crimp deformation of forming layer and blowing powder appear easily. Using the high-speed deflection characteristic of electron beam scanning, before the melting sintering, the metal powder was heated to 900 °C. The preheating process has three advantages, such as improving the conductivity of powder to help the electrons flow out of the build bed, minimizing thermal gradient to reduce the internal stress in the build part and maintaining a uniform temperature in the build part. Presently, EBM has been attracting wide attention because of its ability to be applied to advanced metal alloys, such as cobalt-based alloy [12,13,14], nickel-based alloy [15], titanium and titanium-based alloy [16,17], stainless steels [18,19], etc. [20,21,22].

Due to the uniqueness of the EBM process, for example, directional solidification by scanning electron beam and successive stacking of layered 2D slices, single-crystal like structures can be obtained. Superalloys fabricated by EBM with such microstructure characteristics demonstrate superior mechanical performance. In a recent study of superalloys produced by electron beam melting (EBM), a columnar, [200] textured γ grains with a large number of low angle boundaries were observed [23]. With different angles between the cylindrical axis and Z axis, the products have unique microstructure along with mechanical properties. As reported by Sun et al., the microstructure and high-temperature tensile properties of Inconel 718 (In718) alloys fabricated by EBM demonstrated strong build direction dependence [24]. However, the relation between high temperature oxidation property and build direction has not been investigated yet.

Therefore, in the present paper, three kinds of EBM-In718 alloy specimens with the direction of 0°, 55° and 90° deviation from the Z axis were prepared. The high temperature oxidation behavior of In718 alloys produced by EBM with different building directions was studied for the first time. The association between the anisotropic microstructure and the resultant oxidation performance was clarified in detail.

## 2. Materials and Experiments

### 2.1. Materials

Three kinds of Inconel 718 rods with the dimension of 15 mm in diameter and 85 mm in height varying in building direction were manufactured by an Arcam A2 EBM system (Arcam AB, Mölndal, Sweden) with gas atomized powder. Table 1 lists the detailed chemical composition of Inconel 718 alloy powder. 

**Figure 1:**
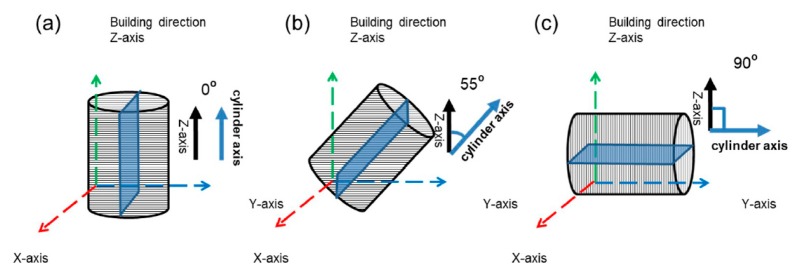
Schematic representation of three-dimensional diagram of electron beam melting (EBM)-In718 alloy columnar specimens with a size of (Φ15 × 85 mm) and the planes used for experiments: (**a**) 0° sample; (**b**) 55° sample and (**c**) 90° sample.

Figure 1 shows the schematic figures of the Inconel 718 rod cylindrical orientations corresponding to the electron-scanning direction and the stacking direction during the EBM process. The specimens were built with the 0°, 55° and 90° deviation angle from the building direction, and are expressed as the 0°, 55° and 90° specimens, separately.

**Figure 2:**
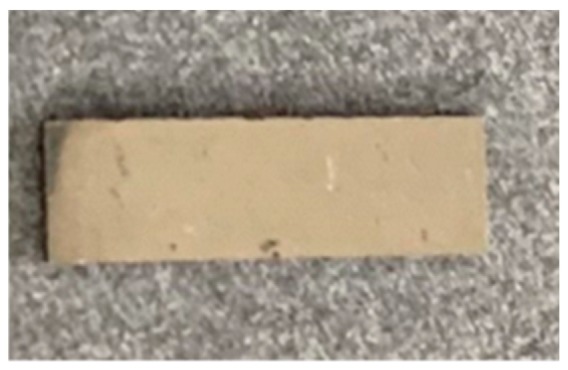
Sheet specimens cut from the middle sections of the Inconel 718 rods.

Figure 2 shows the 5 × 10 × 2 mm^3^ sheet specimens cut from the middle sections of the Inconel 718 rods via wire electrical discharge machining (WEDM, DK7740F, Sihai CNC machine tool factory, Taizhou, China), (the shadow areas in Figure 1). The surface and edges of all specimens were abraded on a series of # 800-2000 grit silicon carbide papers and then polished with alumina suspensions for 15 min. Then, the specimens were cleaned in ethanol by ultrasonic wave cleaner. Finally, all specimens were washed twice using ultra-pure water and dried with an air pump, then put in the electric vacuum drying oven at 70 °C for 2 h. Therefore, the mirror-polished specimens were already prepared for the high temperature oxidation experiments. To characterize the microstructure of the three kinds of specimens, all specimens were polished with OP-S solution for 30 min.

### 2.2. Isothermal Oxidation Test

Isothermal oxidation was conducted at various temperatures 700, 800, 900 and 1000 °C using a muffle furnace in air for 2, 4, 12, 24, 48, 72 and 100 h, respectively. The specimens were placed tilted in a corundum crucible to make sure that the contact area with the crucible is smallest. After exposure, the specimens were taken out of the box furnace along with the crucibles and then rapidly cooled to room temperature. 

### 2.3. Microstructure Characterization

X-ray diffraction (XRD) investigations of the three kinds of specimens before and after the isothermal oxidation tests were performed with a Co anode (λ = 1.79020 Å) in a Bruker D8 Diffraction Advance (Billerica, MA, USA) at room temperature. During the XRD process, each specimen was scanned with a scanning rate of 8°/min from 20° to 80°. The initial grain structures of the specimens were examined using electron backscatter diffraction (EBSD, FEI XL30S, FEI Company, Hillsboro, OR, USA). The EBSD analysis was conducted at step size of 1.5 μm with the accelerating voltage up to 30 kV. From the EBSD results, including inverse polar figure (IPF) map, phase distribution map, image quality (IQ) map, grain size and grain boundary density of all the specimens were obtained using orientation imaging microscopy (OIM) software from EDAX (TSL-OIM 5.0, Tex SEM Laboratories, Provo, UT, USA). The surface morphology and the chemical composition of the three kinds of specimens before and after the isothermal oxidation tests were performed with a NOVA scanning electron microscope (SEM, NOVA NANO SEM 230, FEI Company, Hillsboro, OR, USA) with an Oxford Instruments energy-dispersive X-ray spectroscopy (EDS) detector. The cross-sectioning of the oxidized specimens were performed with a diamond saw. Additionally, postprocessing of the cross-sectional specimens was done with a series of alpha alumina suspensions.

## 3. Results

### 3.1. Alloy Characteristics

**Figure 3:**
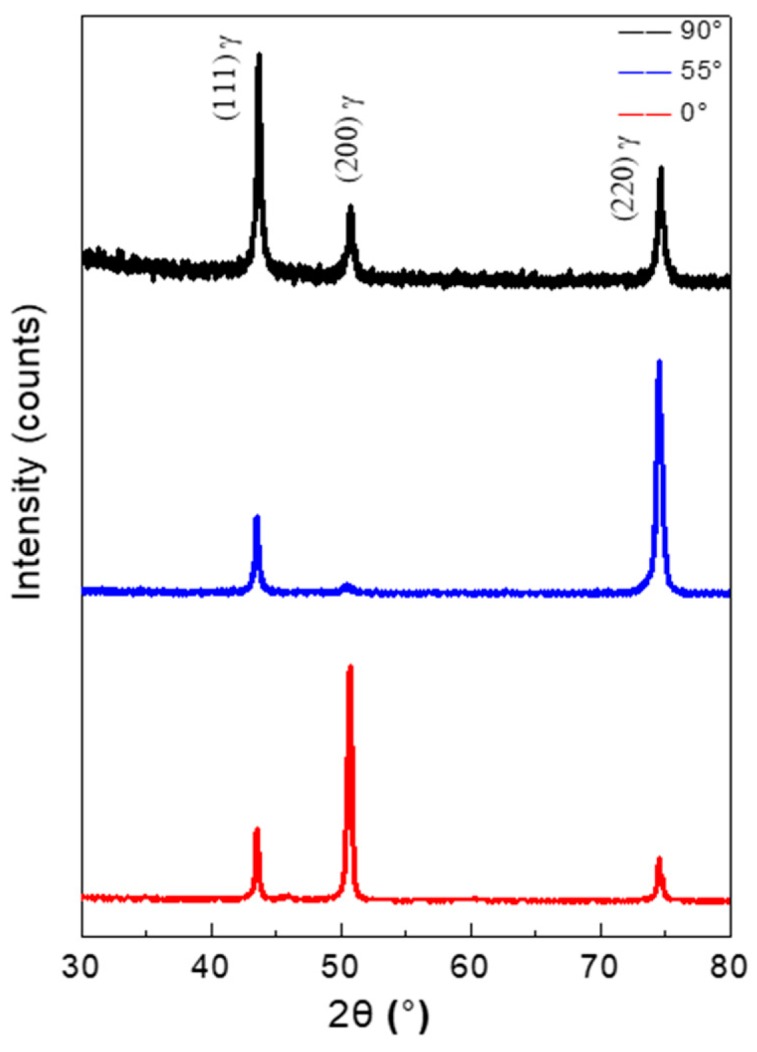
X-ray diffraction (XRD) patterns for the 0°, 55°and 90° specimens of the EBM-In718 alloys.

The XRD patterns of the as-EBM-In718 alloy specimens are exhibited in Figure 3. As the results show, the XRD pattern are indexed, belonging to the face centered cubic (FCC) structure of γ-phase of all the specimens. No additional phase was detected in the XRD results. Furthermore, distinct difference of texture can be observed from the noteworthy variation in peak intensity in various building directions. A strong (100) texture can be seen in the 0° specimen and the 55° specimen reveals a maximum peak at (110) and a weakest peak at (100). What is more, the 90° specimen has a maximum peak at (111), indicating the strong building direction-dependence of the microstructure.

**Figure 4:**
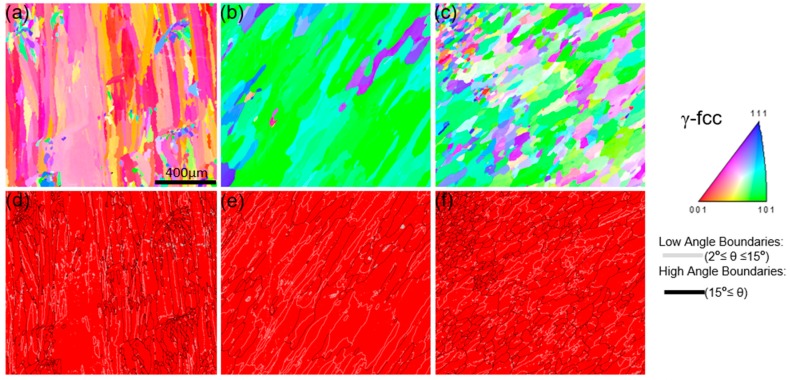
Electron backscatter diffraction (EBSD) inverse polar figure (IPF) maps (**a,b,c**) and image quality (IQ) + phase maps (**d,e,f**) of the EBM-In718 specimens: (a,d) 0° sample; (b,e) 55° sample and (c,f) 90° sample.

**Figure 5:**
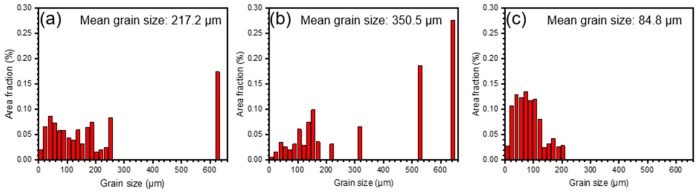
Grain size distributions in the vertical cross-section of EBM-In718 specimens: (**a**) 0° sample; (**b**) 55° sample and (**c**) 90° sample.

**Figure 6:**
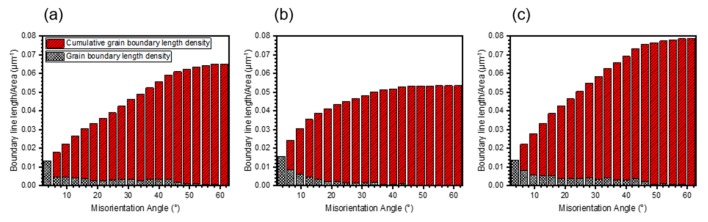
Distributions of grain boundary length density and cumulative grain boundary length density as a function of misorientation angle obtained from EBSD analysis: (**a**) 0° sample; (**b**) 55° sample and (**c**) 90° sample.

As shown in Figure 4a–c, three kinds of specimens demonstrate different grain morphology from the IPF maps. The IPF maps illustrate the strong (001) texture in the 0° sample, (110) texture in 55° sample, and (001), (110) and (111) texture in 90° sample, which are in accord with the XRD results. The image quality (IQ) maps overlapped with the corresponding phase (Figure 4d–f) show the location of the grain boundaries clearly, in which the light grey lines stand for the low angle grain boundaries (LAB) (2° ≤ θ < 15°) and the black ones represent high angle grain boundaries (HAB) (15° ≤ θ). In the three kinds of specimens, a major γ-fcc phase and a large number fraction of LAB are noted in 55° sample. As the grain size distributions (Figure 5) show, the grain size is completely different from one kind of specimen to another. The mean grain size is increased in the order of 90° < 0° < 55°.The calculated grain boundary length density and cumulative grain boundary length density versus misorientation angle are shown in Figure 6. The grain boundary density mainly concentrates on low misorientation angle range (2° to 15°) for all the specimens. In addition, the distributions of grain boundary length density vary with misorientation angle, which illustrate that the grain boundary densities are in an order opposite to the mean grain size of the specimens.

### 3.2. Characterization of Oxide Scales

**Figure 7:**
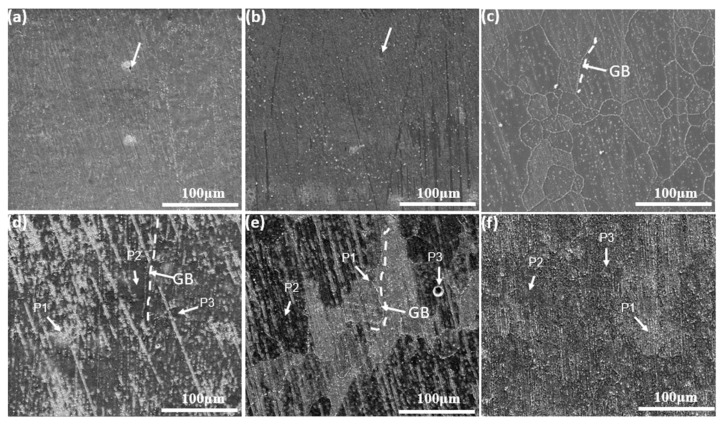
Surface morphology of alloys after oxidation at 700 °C for 4 and 100 h: (**a,d**) 0° sample; (**b,e**) 55° sample; (**c,f**) 90° sample.

After oxidation for less than 4 h at low temperatures (700 °C), light blue is observed in all sample surfaces irrespective of deviation angle. The surfaces turned dark blue after persisted oxidation (more than 24 h). Nevertheless, oxidation at a higher temperature generated darker surfaces, indicating that the composition of the oxide scale depends on the temperature and time of oxidation. Figure 7 shows the surface morphologies of all the specimens oxidized at 700 °C for 4 and 100 h by secondary mode. After oxidation for 4h, the surface of the 90° EBM-In718 alloy specimens (Figure 7c) was much rougher than those of the 0° and 55° specimens (Figure 7a,b). The oxides of 90° specimens were clearly concentrated at grain boundaries (dash lines). Thin and smooth oxide scale can only be observed on 0° and 55° specimens. In addition, the oxide scales of the 0° and 55° specimens (Figure 7d,e) grows not so obviously, while those of the 90° specimens (Figure 7f) became thicker when the oxidation time increased to 100h. From Figure 7d,e, we can see that the oxides were slightly enriched at grain boundaries. At the same time, the grain boundaries of 90° specimens are not distinct. As shown in Figure 7a,b, small pores (indicated by white arrows) appeared on the 0° and 55° specimen surfaces after oxidization at 700 °C for 4 h. These pores on both 0° and 55° specimens were almost invisible when oxidized for 100 h, as shown in Figure 7d,e.

**Figure 8:**
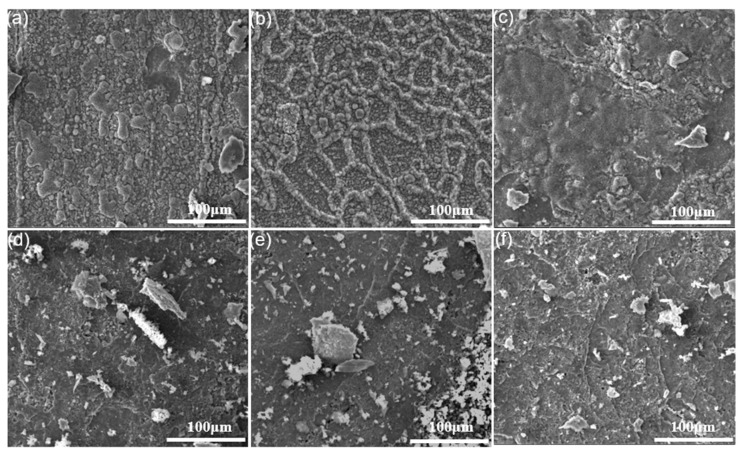
Surface morphology of alloys after oxidation at 1000 °C for 4 and 100 h: (**a,d**) 0° sample; (**b,e**) 55° sample; (**c,f**) 90° sample.

**Figure 9:**
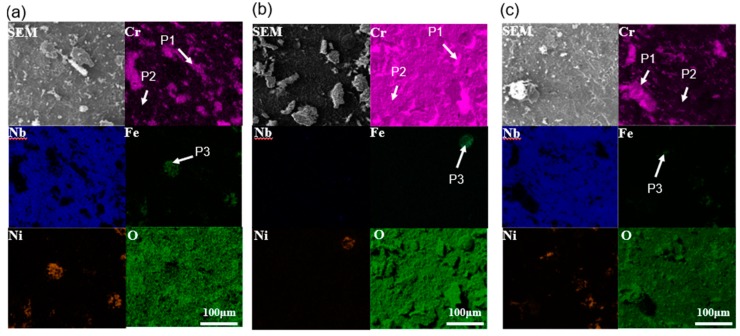
Elemental distribution maps of oxide surface after oxidation at 1000 °C for 100 h: (**a**) 0° sample; (**b**) 55° sample; (**c**) 90° sample.

When the oxidation temperature was up to 1000 °C, the spallation of the oxide scale on all alloys (Figure 8) was more severe than that of the oxide scale at 700 °C (Figure 7). Still, no matter what the oxidation time was, the 90° sample always had a rougher and thicker surface. Figure 9 shows the EDS elemental distribution maps after the specimens oxidized at 1000 °C for 100 h. Contrary to the thin oxide scales of the 0° and 55° specimens oxidized at 700 °C (Figure 7d,e), the specimens oxidized at 1000 °C presented apparent spallation (Figure 9a,b), indicating a reasonably high degradation rate at this temperature. From the EDS results, the oxide scales were generally composed of Cr oxides and Nb oxides.

EDS point analysis identified the compositions of P1 in Figure 7 and Figure 9 as 40–48 at. % O and 25–38 at. % Cr, indicating that the areas consisted of Cr_2_O_3_. At P2, 22–36 at. % O and 35–52 at. % Nb shows that the main composition of the oxide scales was NbO. As for P3, the oxide scales were composed of Ni and Fe oxides. Results of the EDX point analysis at P1, P2, and P3 are listed in Table 2 for all specimens. Based on the chemical compositions results, there was noteworthy Nb segregation, although Ni and Fe were depleted compared to the base metal. Thus, most NbO in P2 was grown underneath the Cr_2_O_3_ layer, which is much more obvious in the 0° and 90° specimens (Figure 7d,f and Figure 9a,c).

**Figure 10:**
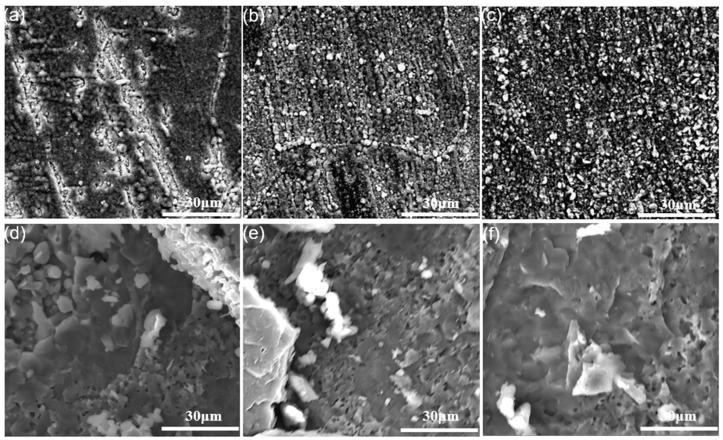
Surface morphology of alloys after oxidation for 100 h at 700 and 1000 °C: (**a,d**) 0° sample; (**b,e**) 55° sample; (**c,f**) 90° sample.

The higher-magnification oxide scales images of the three kinds of specimens oxidized at 700 and 1000 °C for 100 h respectively, are shown in Figure 10. Short columnar oxide grains can be seen on the oxide scales after oxidized at 700 °C. Besides, the size of the oxide grains distributed evenly, while the oxide grains of the 55° specimens were the smallest and the most compact in the three specimens (Figure 10b). Nevertheless, no apparent difference in the morphology of the oxide scale was seen among all the specimens (Figure 10d–f) oxidized at 1000 °C. All specimens were characterized by compact leaflike oxide scales.

### 3.3. Phase Analysis of Oxide Scales

**Figure 11:**
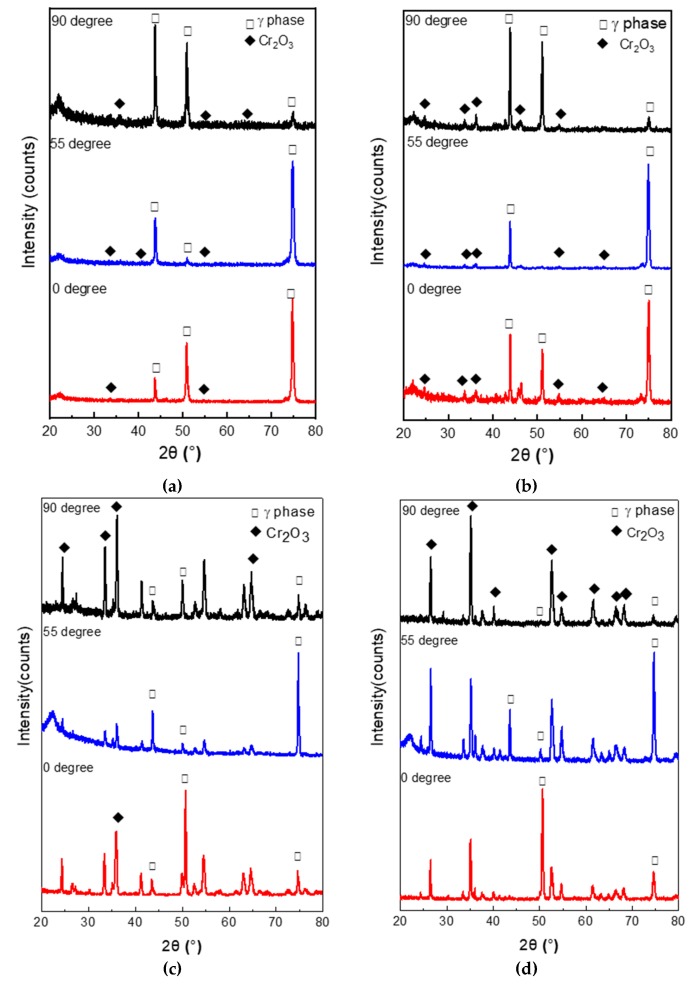
XRD patterns of the three alloys after oxidation at (**a**) 700 °C for 4 h; (**b**) 700 °C for 100 h; (**c**) 1000 °C for 4 h, and (**d**) 1000 °C for 100 h.

The XRD analysis shown in Figure 11a–d presents the oxides of the three kinds of specimens after being oxidized at 700 and 1000 °C for 4 and 100 h, respectively. The results indicate that the oxides formed a protective oxide Cr_2_O_3_, even when the alloy was oxidized only for 4 h, at 700 °C. When the specimens were oxidized for a short time and low temperature, the alloy substrate accounted for the major proportion according to relative peak intensity in the three specimens. Besides, as shown in Figure 11b–d, the higher the temperature and the longer the time, the stronger the intensity of Cr_2_O_3_. Combining the previous EDS results with the XRD analysis, the presence of NbO was not examined by XRD in the 0° and 55° specimens, perhaps owing to the low concentration of NbO underneath the oxide scales or the thick Cr_2_O_3_ scale over the NbO scale.

### 3.4. Cross-Section Profiles

**Figure 12:**
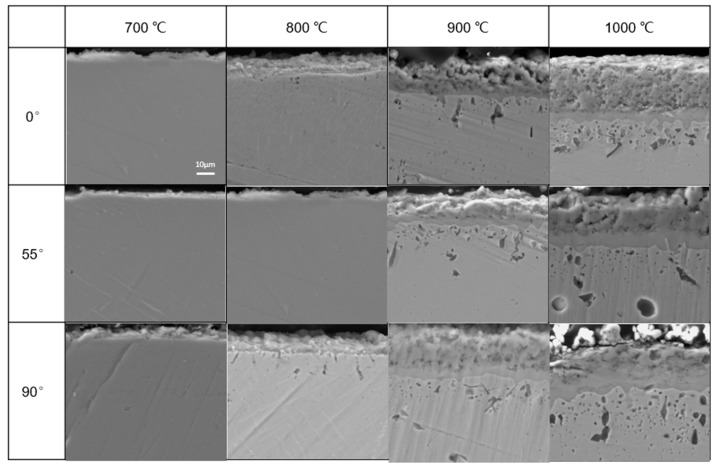
Cross-sectional morphology of EBM-In 718 alloys after oxidation for 100 h at different temperatures.

**Figure 13:**
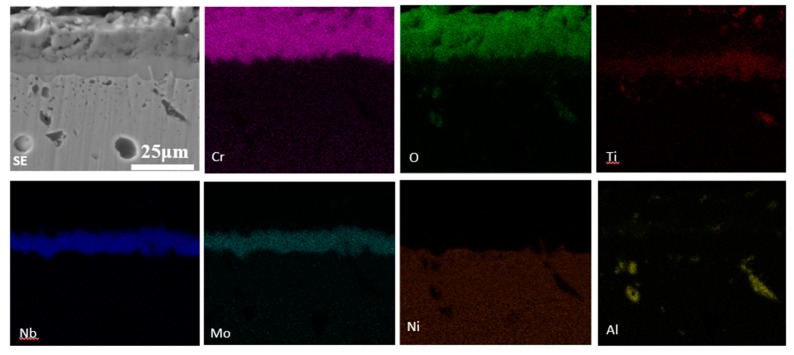
Elemental distribution maps of cross-sectional surface of 55° specimens after oxidation at 1000 °C for 100 h under secondary electrons (SE) mode.

Oxide scale thicknesses of the three kinds of specimens at various temperatures and oxidation times were analyzed by SEM. The SEM images of cross-sectional specimens after oxidation ranging from 700 to 1000 °C for 100 h are shown in Figure 12. The oxide scale defects like cracks and spallation were observed frequently in both 0° specimens and 90° specimens. Furthermore, the formation of defects (cracks or pores) at the interface between the oxide layer and alloy substrate in the two specimens was detected frequently. For 55° specimens, continuous oxide scales without crack were examined, no matter what the oxidation condition was. No apparent separation at the oxide-scale-alloy interface was detected. Figure 13 shows the elemental distribution maps of the cross-sectional 55° specimens under the condition of 1000 °C for 100 h. The outermost layer of oxide scales primarily consisted of Cr oxides, from the XRD results, we could conclude that Cr oxides are a Cr_2_O_3_ film. Additionally, Nb oxides and Mo oxides were present below the Cr_2_O_3_ film.

### 3.5. Oxidation Kinetics

**Figure 14:**
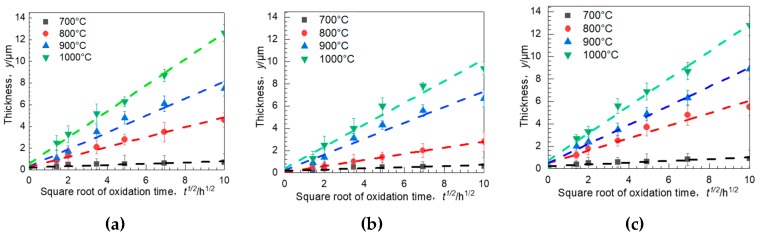
Oxide thickness as a function of oxidation duration for (**a**) 0° sample; (**b**) 55°sample, and (**c**) 90° sample after oxidization at 700, 800, 900, and 1000 °C.

Oxide thickness as a function of oxidation duration for all the specimens are shown in Figure 14. Apparently, the relationship between the Oxide thickness and oxidation temperature/time in our case may conclude with the following equation [25]:

(1) $$\frac{dx}{dt}=\frac{k_{p}}{x}$$

where *x* stands for the thickness of oxide scales, *t* represents the oxidation duration, and *k_p_* means the parabolic rate constant. Comparing to the rapid oxidation in the early time in all conditions, the growth speed of the oxide-scale thickness instantly decelerated. From the oxidation kinetic curves, we can see that when the oxidation temperature is higher by 100 °C, the thickness of oxide scales could grow more than twice, indicating the important influence of the oxidation temperature. Moreover, under the same condition, the oxide thickness of both 0° specimens and 90° specimens were much higher than that of the 55° specimens. According to Equation (1), the following equation can be derived.

(2) $$x=\sqrt{2k_{p}}t^{\frac{1}{2}}$$

**Figure 15:**
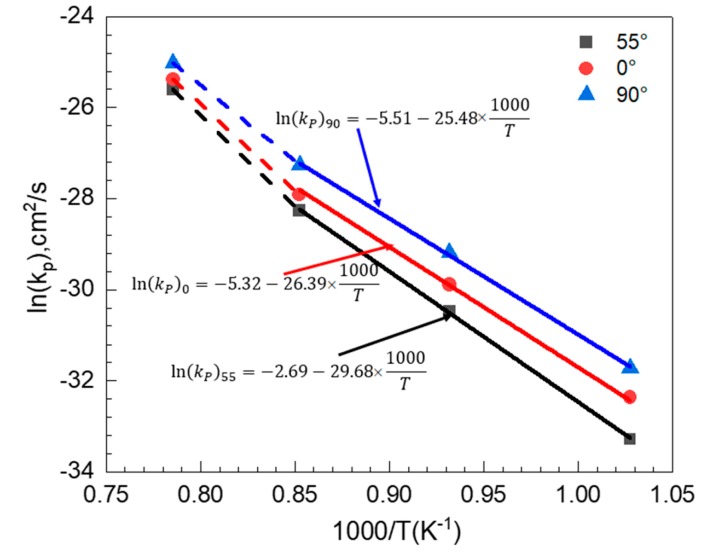
Arrhenius plot for the oxidation of EBM-In718 alloys.

Table 3 lists the values of *k_p_* at each temperature and building direction together with the correlation coefficient value, *r*^2^. The values of *r*^2^ of the values of *k_p_* ranged from 0.90 to 0.99, indicating that the parabolic oxidation law is reasonably applicable to the oxidation behavior. The parabolic rate constant *k_p_* depends on temperature according to the Arrhenius equation [26],

(3) $$k_{p}=\;k_{0}\;\exp\;(-\frac{Q}{RT})k_{p}=\;k0_{\;}\exp\;(-\frac{Q}{RT})$$

where *k*_0_ represents a constant, *Q* stands for the activation energy of oxidation, *T* means the absolute temperature in Kelvin, and *R* is the universal gas constant. Figure 15 shows the Arrhenius plot for the oxidation of EBM-In718 alloys, the activation energy of the oxidation can be calculated by presenting the variation of *k_p_* from the fitting lines. They can be evidently divided into two stages: The high temperature stage (900–1000 °C) and the low temperature stage in the remainder (700–900 °C). For the 0° specimens and 90° specimens, the activation energies were respectively analyzed to be 219.39 and 211.84 kJ/mol when the oxidation temperature was under 900 °C. However, the activation energy of the 55° specimens was higher (246.78 kJ/mol), suggesting that 55° specimens underwent slower oxidation. For the high temperature stage, the activation energy of the specimens is higher but still satisfies the following relation: 90° < 0° < 55°. Hence, the oxide thickness of the EBM-In718 alloys at various temperatures was strongly dependent on the building direction.

## 4. Discussion

It can be concluded from the results that changing the building direction of EBM-In718 alloys has obvious effects on oxidation behavior. The oxidation resistance of EBM-In718 alloys is in the order of 90° < 0° < 55°. To explain the build direction dependence of the oxidation behavior of EBM-In718 alloys, the anisotropies of EBM-In718 alloy in crystal orientation and grain boundary length density with different build directions need to be considered. In addition, the oxide scale defects (cracks and spallation) could be deeply avoided when alloys were built at 55°, particularly after higher oxidation temperature or longer oxidation time.

### 4.1. Oxidation Behavior of EBM-In718 Alloys

The characteristic of the oxide scale in all specimens was greatly influenced by oxidation condition. As Figure 11 shows, the intensity of alloy substrate is extremely weak when the specimens were oxidized at higher temperature and/or longer oxidation time, which indicates the growth of oxide scales. In addition, this phenomenon is in accordance with the color change of the oxide scale, indicating the color change is strictly affected by the growth of the oxide scales. However, the great influence of oxide scale composition on its color cannot be ignored, which is in accordance with the XRD results (Figure 11). From Figure 11, it can be seen that the peak intensities of Cr_2_O_3_ oxides is dissimilar in different oxidation conditions. The high temperature oxidation behavior of In718 alloys has been widely studied for many years [27,28,29,30]. It is reported that the oxidation resistance of a Ni-based system is mainly controlled by oxidation conditions such as the temperature, the time, and the quantity of Cr element [28,31]. When the oxidation temperature is low (700 °C), the initial oxidation may be affected by the simultaneous development of both Cr_2_O_3_ and NbO oxide in the three kinds of EBM-In 718 alloys. Finally, the oxide scales would gradually grow into a Cr_2_O_3_-dominant scale. Consequently, with the Cr_2_O_3_-dominant scale formed by the selective oxidation of Cr, the oxygen partial pressure in the oxide scale comes to be considerably lower than that in air. As a result, the initial oxidation occurred by the simultaneous formation of NiO, Cr_2_O_3_, and MoO_3_ scale. As the oxidation time increased, NiO and MoO_3_ were gradually replaced by Cr_2_O_3_, forming a single-layered Cr_2_O_3_ scale no more than 24 h. When the oxidation temperature was high, Cr_2_O_3_ scales rapidly formed and covered the specimen surface within a short oxidation time. As the EDS mapping analysis of Figure 13 shows, with the oxidation time up to 100 h, a Cr-depleted region formed owing to the decrease in Cr concentration under the oxide scale. The thickness of the Cr_2_O_3_ scale after prolonged oxidation would increase with the higher temperature. As the diffusion coefficient of the Cr element was higher in the grain boundary than that in the alloy substrate, the concentration of Cr in the grain boundary would be considerably lower than that prior to oxidation with the increase in oxidation time. Horita et al. have reported that as a consequence of the rapid thickening of the outermost oxide scale at high temperature, the oxygen diffusion through the scale was greatly hindered [32], as clearly indicated by the decreasing amount of oxygen in the image of O element in Figure 13. From Figure 9, oxide scales formed on the specimen oxidized at 700 and 1000 °C for 100 h were mainly Cr_2_O_3_. 

### 4.2. Effect of Grain Boundary Density on Scale Growth

The grain boundaries are the preferred sites for oxidation [33]. At grain boundaries, the arrangement of atoms is irregular, and the distribution of composition is non-uniform, which make it easy to oxidize. Furthermore, the oxidation behavior was significantly affected by the elements’ diffusion. The diffusion can be divided into two types: Lattice diffusion and grain boundary diffusion. From our present results, the oxide scales were mainly composed of Cr_2_O_3_ and NbO. The growth of Cr_2_O_3_ scales was influenced by Cr diffusion. In this case, the active energy of oxidation can be divided into two stages, which means the Cr diffusion types were different in various temperatures. When oxidation temperature was below 1000 °C, the Cr diffusion was grain boundary diffusion, and the grain boundary length densities of the specimens are in the order of 90° > 0° > 55°. The 55° sample has the smallest grain boundary densities, so it is the hardest for Cr diffusion, which means it is difficult to oxidize. In addition, Nb is difficult for outward transport, so the outward transport of Cr is generally suppressed by the NbO scale formed at the grain boundary. 

**Figure 16:**
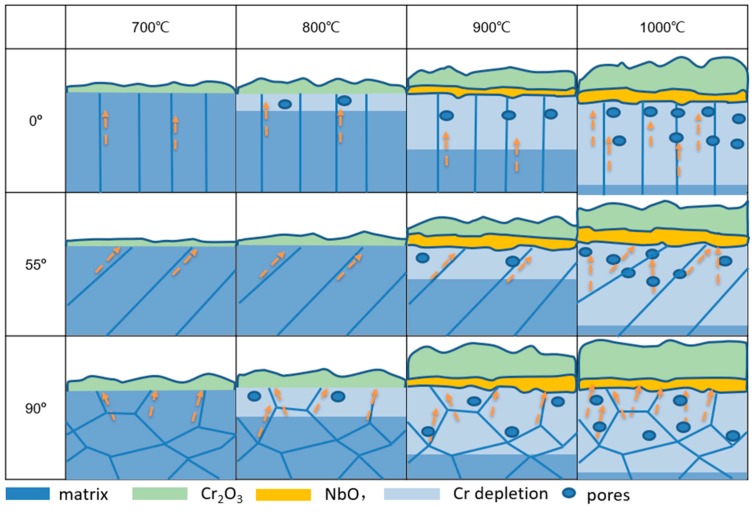
Oxidation mechanisms of EBM-In718 alloys with different build direction in high temperature for 100 h.

On basis of the above discussion, the oxidation mechanisms of EBM-In718 alloys with different build direction are schematically interpreted in Figure 16. The oxidation behavior of EBM-In718 alloys falls into two categories depending on oxidation temperature: Below 1000 °C, 1000 °C.

For all alloys in the low oxidation temperature, the outer layer of oxide scale is the Cr_2_O_3_ layer, meanwhile a thin and continuous NbO layer can be seen under Cr_2_O_3_ layer. The developing of Cr_2_O_3_ is mainly by the transport of Cr atoms along the grain boundaries. Therefore the 55° sample, has the least grain boundaries, and is the most difficult to be oxidized.

When oxidation temperature increases to 1000 °C, the formation of oxide scale is the same as the low temperature situation. The only difference is that the transport of Cr atoms is both along the grain boundaries and bulk grains. Therefore, the oxidation in 1000 °C has no distinct difference.

## 5. Conclusions

The microstructure and high temperature oxidation behavior of Inconel 718 alloys fabricated by EBM in various build directions are systemically studied. Based on the above results, the following conclusions were drawn:

(1) The microstructure of the Inconel 718 alloy fabricated by EBM mainly consists of γ-fcc phase with preferential crystal orientations in various build directions.

(2) The higher the grain boundary length density is, the worse oxidation resistance the alloy shows. The 55° specimen demonstrates the highest oxidation resistance at various temperatures 700, 800, 900 and 1000 °C, owing to its lowest grain boundary length density.

(3) The anisotropy in grain orientation has little effect on the oxidation behavior of the EBM-In718 alloys.

## Figures and Tables

**Table 1:** Chemical composition of Inconel 718 powder (wt. %).

Fe	Cr	Mo	Nb	Ti	Co	Al	C	N	Ni
17.8	19.4	2.97	4.88	0.84	0.10	0.48	0.0355	0.0077	Bal.

**Table 2:** Energy-dispersive X-ray spectroscopy (EDS) point analysis results (wt. %) at points 1–3 for alloy specimens shown in Figure 7 (after oxidation at 700 °C for 4 h and 100 h) and Figure 9 (after oxidation at 1000 °C for 100 h).

Condition	Alloy	P1					P2					P3				
		Ni	Cr	Fe	Nb	O	Ni	Cr	Fe	Nb	O	Ni	Cr	Fe	Nb	O
700 °C, 100 h	0°	19.6	27.3	3.2	1.8	48.1	12.8	13.4	8.8	38.3	26.7	23.3	13.5	15.2	1.6	46.4
	55°	21.9	25.8	9.6	2.1	40.6	14.7	10.2	9.4	40.6	25.1	31.3	6.5	23.3	1.8	37.1
	90°	14.8	33.3	2.6	0.4	48.9	10.9	15.1	8.4	35.4	30.2	22.5	16.3	11.1	1.1	49
1000 °C, 100 h	0°	13.4	32.5	5.3	1.3	47.5	9.5	9.3	6.1	45.3	29.8	27.5	7.6	19.2	1.1	44.6
	55°	15.7	28.8	7.5	1.6	46.4	10.7	8.6	6.5	51.6	22.6	33.1	7.1	24.6	1.5	33.7
	90°	9.2	37.6	3.8	0.9	48.5	8.8	10.1	5.5	39.2	36.4	25.4	10.2	18.3	0.7	45.4

**Table 3:** Square root of parabolic rate constant, correlation coefficient, and activation energy of EBM-In718 alloys.

Parabolic Constant $\sqrt{2kp}\mathrm{cm}\;s^{-1/2},\;(r^{2})\;$	700 °C	800 °C	900 °C	1000 °C	Q kJ/mol
0°	8.79 × 10^−15^ (0.9014)	8.89 × 10^−14^ (0.9986)	9.07 × 10^−13^ (0.9706)	2.01 × 10^−12^ (0.9645)	219.39
55°	3.88 × 10^−15^ (0.9786)	6.54 × 10^−14^ (0.9801)	7.04 × 10^−13^ (0.9687)	1.34 × 10^−12^ (0.9927)	246.78
90°	1.68 × 10^−14^ (0.9543)	2.11 × 10^−13^ (0.9559)	1.45 × 10^−12^ (0.9914)	3.11 × 10^−12^ (0.9879)	211.84
